# Pseudosymmetry in Tetragonal Perovskite SrIrO_3_ Synthesized under High Pressure

**DOI:** 10.1021/acsaelm.4c01214

**Published:** 2024-08-30

**Authors:** Haozhe Wang, Alberto de la Torre, Joseph T. Race, Qiaochu Wang, Jacob P. C. Ruff, Patrick M. Woodward, Kemp W. Plumb, David Walker, Weiwei Xie

**Affiliations:** † Department of Chemistry, Michigan State University, East Lansing, Michigan 48824, United States; ‡ Department of Physics, Brown University, Providence, Rhode Island 02912, United States; § Department of Chemistry and Biochemistry, The Ohio State University, Columbus, Ohio 43210, United States; ∥ Cornell High Energy Synchrotron Source, Cornell University, Ithaca, New York 14853, United States; ⊥ Lamont Doherty Earth Observatory, Columbia University, Palisades, New York 10964, United States

**Keywords:** High-pressure synthesis, Iridates, Phase transition, Mott insulator, Metallic oxides

## Abstract

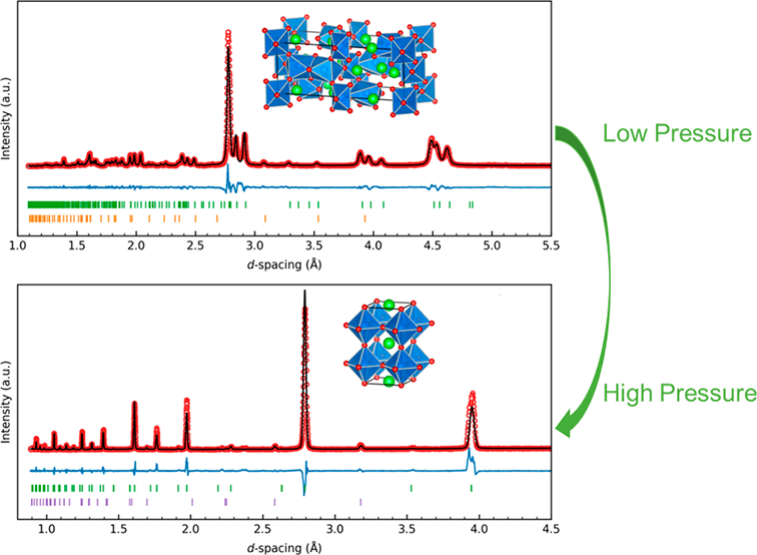

In this study, we report a tetragonal perovskite structure of SrIrO_3_ (*P*4/*mmm*, *a* = 3.9362(9) Å, *c* = 7.880(3) Å) synthesized at 6 GPa and 1400 °C, employing the ambient pressure monoclinic SrIrO_3_ with distorted 6*H* structure as a precursor. The crystal structure of tetragonal SrIrO_3_ was evaluated on the basis of single-crystal and powder X-ray diffraction. A cubic indexing was observed, which was attributed to overlooked superlattice reflections. Weak fractional peaks in the H and K dimensions suggest possible structure modulation by oxygen defects. Magnetization study reveals weak paramagnetic behavior down to 2 K, indicative of the interplay between spin–orbit coupling, electron correlations, and the crystal electric field. Additionally, measurements of electrical resistivity display metallic behavior with an upturn at about 54 K, which is ascribed to weak electron localization and possible structural defects.

## Introduction

Iridates are an ideal system for investigating the intriguing quantum physics arising from the significant spin–orbit coupling (SOC). The exotic electronic quantum phenomena primarily arise from the interplay between the robust SOC and other fundamental interactions, which have comparable energy scales, including crystal electric field and electron correlations, leading to competition between them. The SOC is typically negligible in the 3*d* transition metal systems. However, in the case of 5*d* transition metal compounds, such as the iridates, the SOC plays a crucial role in the observed physics behaviors, for example, superconductivity,^1^ Kitaev quantum spin liquid,^2^ Weyl semimetal behavior,^3,4^ quantum criticality,^5,6^ and quantum Hall effects.^7^ Among iridate compounds, the Ruddlesden–Popper phases Sr_*n*+1_Ir_*n*_O_3*n*+1_, specifically, Sr_2_IrO_4_ (*n* = 1) and Sr_3_Ir_2_O_7_ (*n* = 2), have been intensively studied for novel physics with strong SOC on Ir sites.^8−12^ In Sr_2_IrO_4_ and Sr_3_Ir_2_O_7_, [IrO_6_] octahedra arrange along the *ab*-plane, forming the two-dimensional patterns. Conversely, owing to the enhanced interlayer hopping induced by the three-dimensional continuous arrangement of [IrO_6_] octahedra, in comparison with Sr_2_IrO_4_ and Sr_3_Ir_2_O_7_, which are insulators with canted antiferromagnetic ordering, SrIrO_3_ (*n* = ∞) exhibits a paramagnetic semimetal ground state.^13,14^ Thus, SrIrO_3_ is positioned in proximity to a multiphase boundary where the transitions of metal–insulator and magnetic ground states occur. These transitions are governed by the combination of multiple interactions, resulting in a complex interplay of competing effects.^13^

Instead of the bulk, most current SrIrO_3_ studies focus on constructing heterostructure thin films based on SrIrO_3_ and other ABO_3_ perovskite oxides (for example, CaMnO_3_) using molecular beam epitaxy along particular crystallographic orientations, such as the [111] direction and the [001] direction.^15−18^ The component layers require thickness at the unit cell scale and lattice quality at the atomic level, which sets a big challenge in sample preparation. On the other hand, the high-pressure and high-temperature synthesis provides a new approach to novel materials.^19^ Recently, the non-centrosymmetric Sr_2_IrO_4_ was first discovered at 6 GPa and 1400 °C,^20^ which further inspires us to investigate SrIrO_3_ under high-pressure and high-temperature conditions.

Herein, we report the synthesis, structural characterization, and magnetic and electrical resistivity study on the tetragonal SrIrO_3_ synthesized at 6 GPa and 1400 °C for 3 h (serial number TT-1446). Employing single-crystal and powder X-ray diffraction (XRD), the tetragonal symmetry was carefully examined, excluding the possibility of pseudo cubic symmetry observed. Moreover, weak fractional peaks with the tetragonal space group in the H and K dimensions in the reciprocal space suggest possible structure modulation by oxygen defects. The magnetization study of tetragonal SrIrO_3_ shows weak paramagnetic behavior down to 2 K. Furthermore, our electrical resistivity measurement demonstrates a metallic property with a resistivity upturn at ∼54 K due to weak electron localization and possible structural defects in the presence of SOC.

## Experimental Section

### High-Pressure Synthesis

The high-pressure synthesis was conducted using a Walker-type^21^ multianvil apparatus (MA) at Lamont-Doherty Earth Observatory. The starting material was the as-synthesized ambient pressure SrIrO_3_ phase, which was prepared by thoroughly mixing the materials SrCO_3_ and Ir, subsequently heating them to 900 °C for 12 h, then regrinding and reannealing at 1000 °C for 72 h.^22^ The sample was kept at 120 °C overnight to remove moisture before high-pressure experiments. The sample was then loaded in a platinum capsule inside an Al_2_O_3_ crucible that was inserted into a Ceramacast 646 octahedral pressure medium lined on the inside with a LaCrO_3_ heater and kept at 6 GPa and 1400 °C for 3 h before quenching to room temperature and then decompressed to ambient pressure overnight (serial number TT-1446). The pressure applied in press was calibrated using the phase transitions of bismuth at room temperature. To ensure accurate temperature readings, thermocouples are positioned as close as possible to the sample for direct temperature measurement.

### Chemical Composition Determination

The recovered high-pressure product was examined for purity and homogeneity using a Zeiss Sigma field emission scanning electron microscope (SEM) with an Oxford INCA PentalFETx3 energy-dispersive spectroscopy (EDS) system (model 8100). An accelerating voltage of 20 kV was employed for imaging and analysis.

### Phase Analysis by Powder X-ray Diffraction

The phase identity and purity were examined using a Bruker Davinci powder X-ray diffractometer with Cu K_α_ radiation (λ = 1.5406 Å). Room-temperature measurements were carefully performed with a step size of 0.010° at a scan speed of 5.00 s/step over a Bragg angle (2θ) range of 5–120°.

### Home Laboratory Single-Crystal X-ray Diffraction

The room-temperature crystal structure was determined using a Bruker D8 Quest Eco single-crystal X-ray diffractometer equipped with Mo K_α_ radiation (λ = 0.7107 Å) with an ω of 2.0° per scan and an exposure time of 10 s per frame. The SHELXTL package with the direct methods and full-matrix least-squares on the *F*^2^ model was used to determine the crystal structure.^23,24^

### Synchrotron Single-Crystal X-ray Diffraction

The experiments were carried out on the QM^2^ beamline at the Cornell High Energy Synchrotron Source (CHESS). The sample was affixed to a Kapton mount using GE varnish, and all data were collected at room temperature. We used an X-ray energy of 18 keV (0.6888 Å wavelength), and data were collected with a Pilatus 6M photon counting detector operating at a 10 Hz frame rate while continuously rotating the sample angle (psi) through 360° (gives one image every 0.1 degree). We combined scans taken with the sample chi angles of 0°, 90°, and 100° in order to fill in gaps in the detector and reduced the data using a cubic unit cell, lattice parameters 3.937 Å.

### Physical Properties Measurement

Temperature and field-dependent magnetization and electrical resistivity measurements were performed with a Quantum Design DynaCool physical property measurement system (PPMS) at a temperature range of 1.8–300 K and applied fields up to 9 T using 11.0 mg of sample in total. Electrical resistivity measurements were conducted with a four-probe method using platinum wires on a polycrystalline sample with the dimensions of 1.0 × 0.8 × 0.5 mm.

## Results and Discussion

### Crystal Structure Determination and Phase Analysis

**Figure 1:**
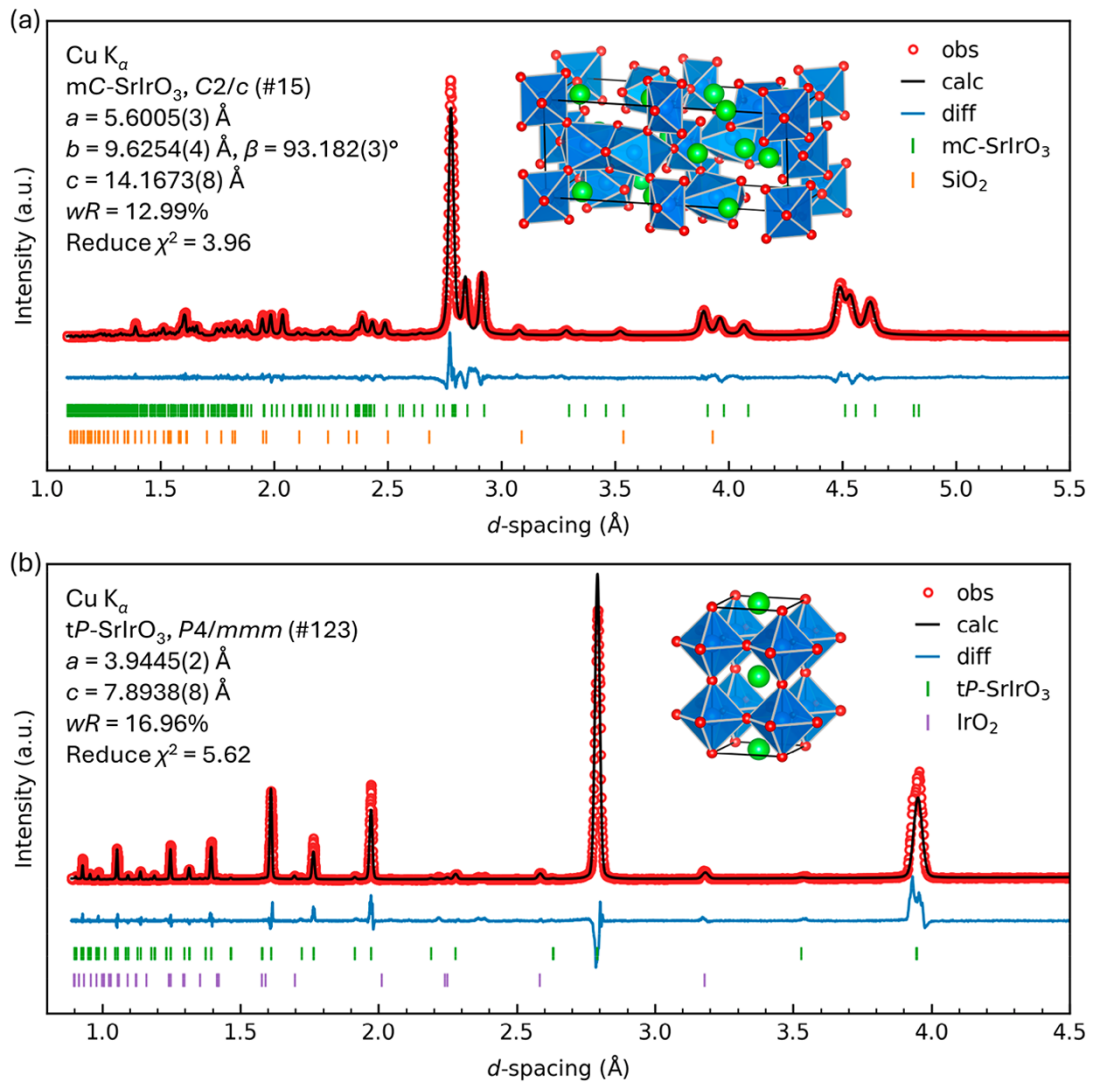
Powder XRD pattern and Rietveld refinement of (a) ambient pressure m*C*-SrIrO_3_ and (b) high-pressure t*P*-SrIrO_3_ synthesized at 6 GPa and 1400 °C. Bragg peak positions of each phase are represented by vertical tick marks. The crystal structures are also shown. Sr, green; Ir, blue; O, red.

The ambient pressure SrIrO_3_ (m*C*-SrIrO_3_) crystallizes in a monoclinic distortion of the hexagonal BaTiO_3_-type structure. Under pressure, an orthorhombic perovskite SrIrO_3_ (o*P*-SrIrO_3_) in the GdFeO_3_-type structure was reported at above 2 GPa at 1650 °C and above 5 GPa at 700 °C.^25^ Here we performed our high-pressure synthesis at 6 GPa and 1400 °C. Prior to pressurization, the precursor phase, as-synthesized m*C*-SrIrO_3_, was confirmed by powder XRD, with the pattern and Rietveld refinement presented in Figure 1a. Around 7% SiO_2_ (by weight) was added as an impurity phase to account for peaks that could not be indexed to m*C*-SrIrO_3_ (refer to Tables S1–S4 for details). Figure 1b shows the powder XRD pattern of our recovered product, where diffraction peak positions clearly illustrate the appearance of a new phase after high-pressure and high-temperature treatment.

**Figure 2:**
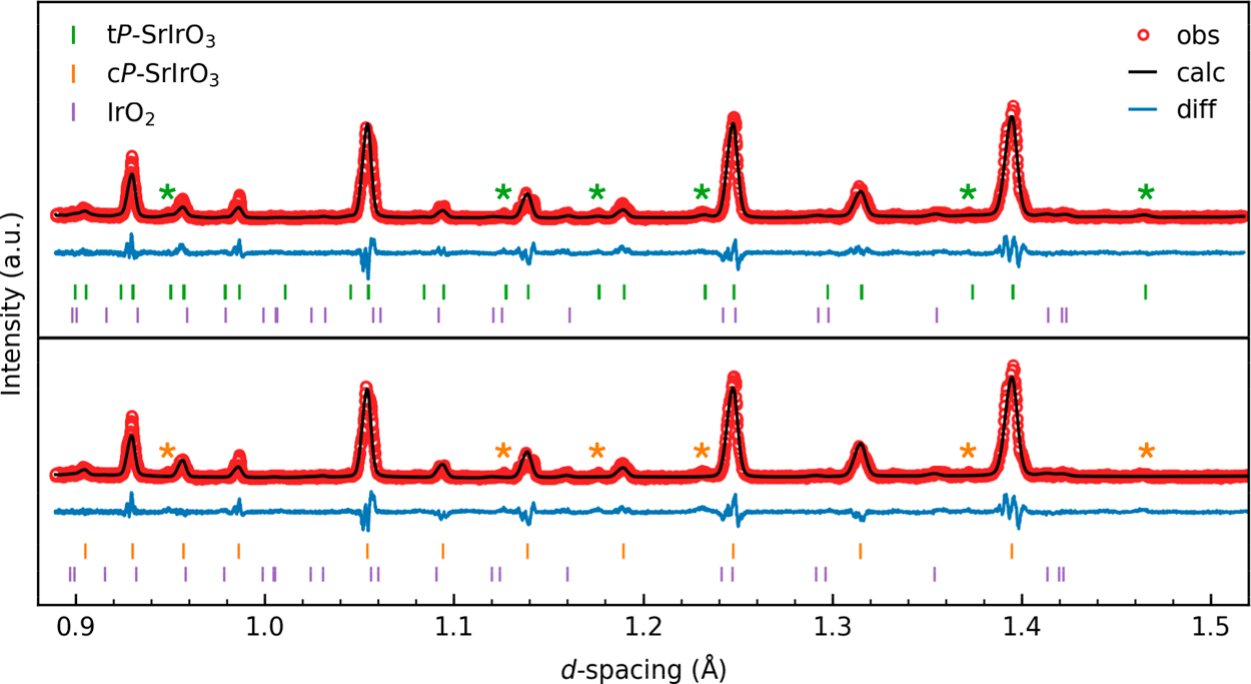
Zoom-in powder XRD pattern of the recovered product and Rietveld refinement using t*P*-SrIrO_3_ and c*P*-SrIrO_3_ models. Bragg peak positions of each phase are represented by vertical tick marks. The crystal structures are also shown. Sr, green; Ir, blue; O, red. Stars indicate diffraction peaks for which the c*P*-SrIrO_3_ model failed to account in comparison to t*P*-SrIrO_3_.

To determine the crystal structure of the recovered product as well as initialize Rietveld refinements of the powder XRD pattern, single-crystal XRD experiments were first completed in a home laboratory. Limited by poor data quality, two structure models were proposed. One is a cubic perovskite structure (*a* = 3.9403(6) Å) with the space group *Pm*3̅*m* (#221), denoted as c*P*-SrIrO_3_; the other is a tetragonal structure with a *c* lattice parameter twice that of the others (*a* = 3.9362(9) Å, *c* = 7.880(3) Å, t*P*-SrIrO_3_) in *P*4/*mmm* (#123). The cubic structure model was slightly preferred. Crystallographic data and our single-crystal XRD refinement details are summarized in Tables S5–S8. Figure S1 provides regenerated reciprocal lattice planes from single-crystal XRD based on these two structure models. Additionally, homogeneous chemical element distribution of the recovered product was confirmed by SEM and EDS analysis on a single-crystal sample (Figures S2 and S3). c*P*-SrIrO_3_ and t*P*-SrIrO_3_ were then taken as starting parameters for the Rietveld refinements. Considering the inclusion of about 3% IrO_2_ (by weight) as a secondary phase, both structure models yielded reasonable refinement parameters (details provided in Tables S9–S12). A minor difference was noted between these two models, specifically within the *d*-spacing range of 0.9–1.5 Å. Unlike t*P*-SrIrO_3_, c*P*-SrIrO_3_ failed to address extra weak but clearly resolved diffraction peaks, as presented in Figure 2, suggesting lower symmetry. The reported o*P*-SrIrO_3_ (*Pbnm*, *a* = 5.5909(1) Å, *b* = 7.8821(1) Å, *c* = 5.5617(1) Å)^22,26^ model was also evaluated; however, the absence of diffraction peaks indicative of orthorhombic symmetry excludes its possibility as the optimized structure model (details shown in Figure S4, Tables S13 and S14).

**Figure 3:**
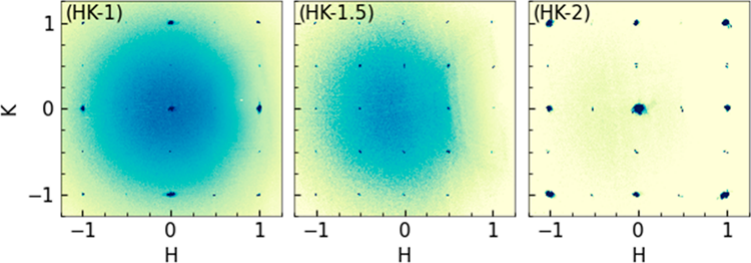
Regenerated reciprocal lattice planes, (HK-1), (HK-1.5), and (HK-2), obtained from data reduction of our synchrotron single-crystal XRD using cubic symmetry and the unit cell of c*P*-SrIrO_3_.

To further assess the accuracy of the cubic indexing suggested by our in-house single-crystal XRD analysis for the crystal structure of our recovered product, we carried out synchrotron single-crystal XRD experiments at the CHESS. For a better comparison, we reduced our data using cubic symmetry and the unit cell of the c*P*-SrIrO_3_ structure model. Figure 3 provides the regenerated reciprocal lattice planes (HK-1), (HK-1.5), and (HK-2). The clearly resolved, relatively intense peaks on the (HK-1.5) plane corroborate the unit cell doubling along the *c*-axis, aligning with our t*P*-SrIrO_3_ structure model. Moreover, weaker fractional diffraction peaks in the H and K dimensions suggest possible structure modulation due to oxygen defects in t*P*-SrIrO_3_. Furthermore, the satellite peaks along the (HK-2) plane indicate the structure is incommensurate, which is probably caused by modulation of the O defects’ modulation. The single crystal may indeed have twinning with the two twinning components related by a 90-degree rotation along the *c*-axis. This twinning law will leave the main reflections invariant (tetragonal) but relates the satellites along the H direction to those along the K direction.

**Figure 4:**
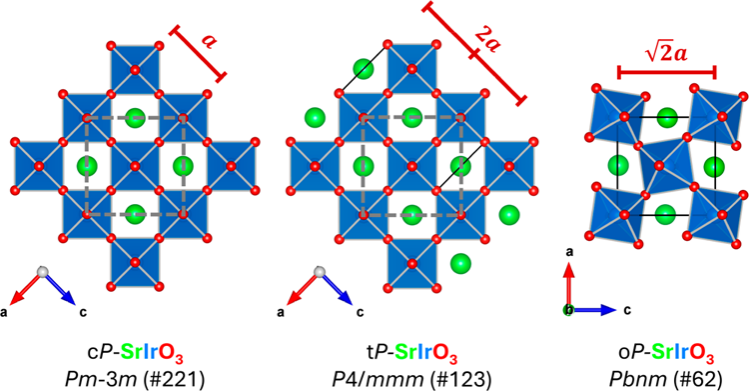
Crystal structure and lattice parameter comparison of c*P*-SrIrO_3_, t*P*-SrIrO_3_, and o*P*-SrIrO_3_. Sr, green; Ir, blue; O, red.

Following the discussion, we identified the optimized structure model of our recovered product as t*P*-SrIrO_3_ in space group *P*4/*mmm* (#123) with the unit cell parameters *a* = *b* = 3.9362(9) Å and *c* = 7.880(3) Å. Figure 4 presents a comparative analysis of the crystal structures and lattice parameters of c*P*-SrIrO_3_, t*P*-SrIrO_3_, and o*P*-SrIrO_3_. Overall, t*P*-SrIrO_3_ shows a ∼4.0% volume compression per chemical formula compared to that of m*C*-SrIrO_3_ under ambient pressure. The average Sr–O and Ir–O bond lengths measure 2.77(4) Å and 1.969(1) Å, respectively. The asymmetric Ir–O distances in octahedral IrO_6_ indicate the existence of polar local distortions. The phase was quenched from high-pressure and high-temperature synthesis and thus can be metastable under ambient conditions. The external pressure is a possible reason for the polar local distortion to be stabilized. Unlike common observations in ambient and high-pressure SrIrO_3_^22,26^ and Sr_2_IrO_4_,^20,27^ the absence of [IrO_6_] octahedra rotation, tilt, and oxygen site disorder indicate potential linear Ir–O–Ir exchange interactions in this t*P*-SrIrO_3_.^28,29^

### Magnetization: Temperature-Dependent Paramagnetism

**Figure 5:**
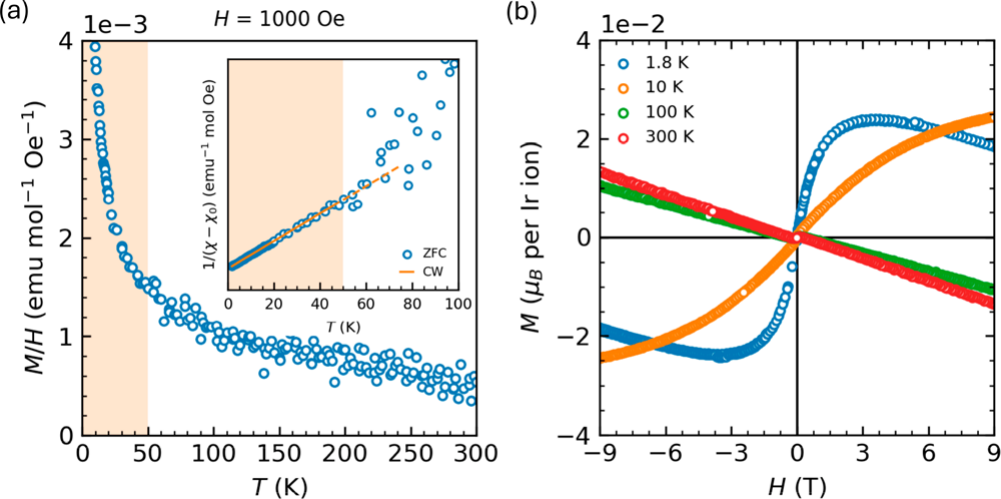
Magnetization of tetragonal SrIrO_3_ synthesized under high pressure. (a) Magnetic susceptibility measured under ZFC mode at 1000 Oe. Inset, ZFC data fitted by the Curie–Weiss model. (b) Field-dependent magnetization at 1.8, 10, 100, and 300 K.

Similar to monoclinic SrIrO_3_ and orthorhombic SrIrO_3_, a paramagnetic susceptibility was observed in tetragonal SrIrO_3_, as displayed in Figure 5a. There is no obvious split between zero-field-cooled (ZFC) and field-cooled (FC) modes (see Figure S5 for details). The initial examination of the data highlighted the small value of positive magnetic susceptibility and its noisy temperature dependence, especially above 50 K. The magnetic susceptibility below 50 K was fitted to the modified Curie–Weiss law (eq 1):
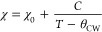
1where θ_cw_ is the Curie temperature, χ_0_ is the temperature-independent magnetic susceptibility, and *C* is the Curie constant. The data were well described by the Curie–Weiss model down to 2 K, yielding a θ_cw_ of −0.01(1) K and an effective magnetic moment per Ir ion, μ_eff_, of 0.494(2) μ_B_. The temperature-independent magnetic susceptibility χ_0_ is equal to 8.9(1) × 10^–4^ emu/mol, which is larger than that of the reported orthorhombic SrIrO_3_.

Figure 5b shows the field-dependent magnetization of tetragonal SrIrO_3_. At 300, 100, and 1.8 K, a clear diamagnetic contribution was observed, becoming significant at high magnetic fields. However, at 10 K, the field-dependent magnetization exhibited unusual behavior, indicating that a standard Curie–Weiss model is insufficient to describe the magnetization behavior of this system. Considering that tetragonal SrIrO_3_ behaves as a metal above 50 K (see our electrical resistivity data below), the magnetic susceptibility of nonlocalized conduction electrons should be taken into account. Then another possible interpretation of the magnetization data arises from itinerant magnetism due to intense electron–electron correlations. Consequently, monotonically increasing temperature-dependent magnetization is not always expected in various fields. This can explain the weak magnetization observed and the small fitted effective moment. Note that different contributions to magnetization are not mutually exclusive. Below 50 K, the localization of electrons was enhanced, and a possible Curie–Weiss paramagnetism arises from the local moment. The more accurate magnetic structure and overall spin dynamics should be determined through further experiments.

### Electrical Resistivity: Metal with a Low-Temperature Upturn

**Figure 6:**
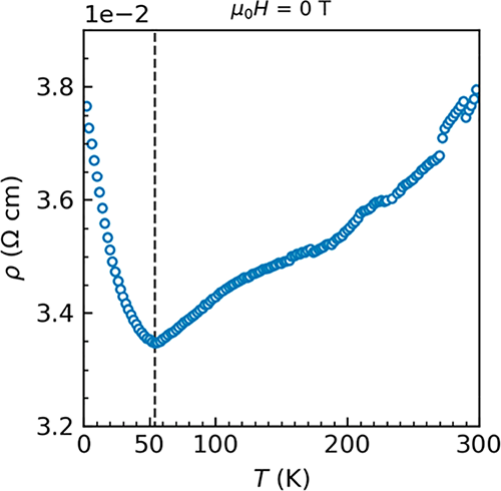
Temperature-dependent electrical resistivity of tetragonal SrIrO_3_ at μ_0_*H* = 0 T. An upturn was observed at ∼54 K.

Figure 6 illustrates the temperature-dependent electrical resistivity of tetragonal SrIrO_3_ from 2 to 300 K, noting a room-temperature resistivity of 0.038(1) Ω cm. The resistivity data show metallic behavior with an upturn at 54(2) K, a feature also reported in bulk orthorhombic SrIrO_3_^22^ and monoclinic SrIrO_3_ thin film systems,^15,16,18^ typically associated with a metal–insulator transition. Note that in the whole temperature range studied, the electrical resistivity only changed from 0.038 to around 0.033 Ω cm, with approximately 13% at maximum. Suggested by magnetization, a possible explanation for the resistivity upturn involves weak electron localization, attributable to intense electron–electron correlations, and possible structural defects in the polycrystalline sample.^30^ In iridates, Ir 5*d* orbitals are highly extended in space and overlap with oxygen *p* orbitals, which induce interlayer hopping. Different from Sr_2_IrO_4_ and Sr_3_Ir_2_O_7_ with two-dimensional [IrO_6_] interlayer hopping, SrIrO_3_, with the enhanced interlayer hopping induced by the [IrO_6_] three-dimensionality, becomes, consequently, a metal at room temperature.^10,31^ From the electronic state point of view, the bands that cross through the Fermi surface are half-filled and largely split. At low temperatures, electron localization is enhanced and then the gap opens, contributing to the resistivity upturn. Figure S6 provides our measurements in different sequences of cooling and warming, confirming the low-temperature resistivity upturn.

## Conclusion

In conclusion, we reported the new tetragonal perovskite SrIrO_3_ (*P*4/*mmm*, *a* = 3.9362(9) Å, *c* = 7.880(3) Å) synthesized at 6 GPa and 1400 °C. The tetragonal crystal structure was determined based on single-crystal and powder XRD results with exclusion of pseudocubic symmetry. Weak fractional peaks in the H and K dimensions suggest possible structure modulation by oxygen defects. Magnetization study indicated weak paramagnetic behavior down to 2 K, with a small fitted effective magnetic moment of 0.494(2) μ_B_, resulting from itinerate electrons. Moreover, an electrical resistivity study demonstrated a metallic property with an upturn at 54(2) K, attributed to weak electron localization and structural defects. This tetragonal SrIrO_3_ provides another example of how high pressure can induce novel physical properties in quantum materials.

## Supporting Information Available

Regenerated reciprocal lattice planes of t*P*-SrIrO_3_, c*P*-SrIrO_3_, and o*P*-SrIrO_3_; SEM and EDS analysis of a single-crystal sample; zoom-in powder XRD Rietveld refinement of t*P*-SrIrO_3_ and o*P*-SrIrO_3_; magnetic susceptibility of t*P*-SrIrO_3_; electrical resistivity of t*P*-SrIrO_3_ in different cooling and warming modes; structure parameters from powder XRD Rietveld refinement of m*C*-SrIrO_3_, m*C*-SrIrO_3_+SiO_2_, t*P*-SrIrO_3_+IrO_2_, c*P*-SrIrO_3_+IrO_2_, and o*P*-SrIrO_3_+IrO_2_; crystallographic data and single-crystal XRD refinement of t*P*-SrIrO_3_ and c*P*-SrIrO_3_; atomic coordinates and equivalent isotropic atomic displacement parameters (PDF)
