# Supplementary material for: Pseudosymmetry in Tetragonal Perovskite SrIrO3 Synthesized under High Pressure
Source: ACS Appl Electron Mater. 2024 Aug 30;6(9):6820–5. doi: 10.1021/acsaelm.4c01214 (PMC11425847; doi:10.1021/acsaelm.4c01214)
Supplement: Supplementary file 1 — el4c01214_si_001.pdf [file el4c01214_si_001.pdf]

Supporting Information

Pseudosymmetry in Tetragonal Perovskite $SrIrO_3$ Synthesized under High Pressure

Haozhe Wang[1], Alberto de la Torre[2], Joseph T. Race[3], Qiaochu Wang[2], Jacob P. C. Ruff[4], Patrick M. Woodward[3], Kemp W. Plumb[2], David Walker[5], Weiwei Xie[1]*

1. Department of Chemistry, Michigan State University, East Lansing, MI, 48824 USA
2. Department of Physics, Brown University, Providence, RI, 02912 USA
3. Department of Chemistry and Biochemistry, The Ohio State University, Columbus, OH, 43210 USA
4. Cornell High Energy Synchrotron Source, Cornell University, Ithaca, NY, 14853 USA
5. Lamont Doherty Earth Observatory, Columbia University, Palisades, NY, 10964 USA

* Email: xieweiwe@msu.edu

Table of Contents

Figure S1 Regenerated reciprocal lattice planes of t*P*-$SrIrO_3$, c*P*-$SrIrO_3$, and o*P*-$SrIrO_3$ S2
Figure S2 SEM and EDS analysis of single crystal $SrIrO_3$ S3
Figure S3 EDS mapping analysis of single crystal $SrIrO_3$ S4
Figure S4 Zoom-in powder XRD Rietveld refinements of t*P*-$SrIrO_3$ and o*P*-$SrIrO_3$ S5
Figure S5 Temperature-dependent magnetic susceptibility of t*P*-$SrIrO_3$....................................S6
Figure S6 Temperature-dependent electrical resistivity of t*P*-$SrIrO_3$ S7

Table S1 Structure parameters from powder XRD Rietveld refinement of m*C*-$SrIrO_3$ S8
Table S2 Atomic coordinates and equivalent isotropic atomic displacement parameters S8
Table S3 Structure parameters from powder XRD Rietveld refinement of m*C*-$SrIrO_3$ + SiO_2 ...S9
Table S4 Atomic coordinates and equivalent isotropic atomic displacement parameters S9
Table S5 Crystallographic data and single crystal XRD refinement of t*P*-$SrIrO_3$.....................S10
Table S6 Atomic coordinates and equivalent isotropic atomic displacement parameters S10
Table S7 Crystallographic data and single crystal XRD refinement of c*P*-$SrIrO_3$ S11
Table S8 Atomic coordinates and equivalent isotropic atomic displacement parameters S11
Table S9 Structure parameters from powder XRD Rietveld refinement of t*P*-$SrIrO_3$ + IrO_2 ....S12
Table S10 Atomic coordinates and equivalent isotropic atomic displacement parameters S12
Table S11 Structure parameters from powder XRD Rietveld refinement of c*P*-$SrIrO_3$ + IrO_2..S13
Table S12 Atomic coordinates and equivalent isotropic atomic displacement parameters S13
Table S13 Structure parameters from powder XRD Rietveld refinement of o*P*-$SrIrO_3$ + IrO_2 .S14
Table S14 Atomic coordinates and equivalent isotropic atomic displacement parameters S14

Figure S1 Regenerated reciprocal lattice planes of t*P*-$SrIrO_3$, c*P*-$SrIrO_3$, and o*P*-$SrIrO_3$.

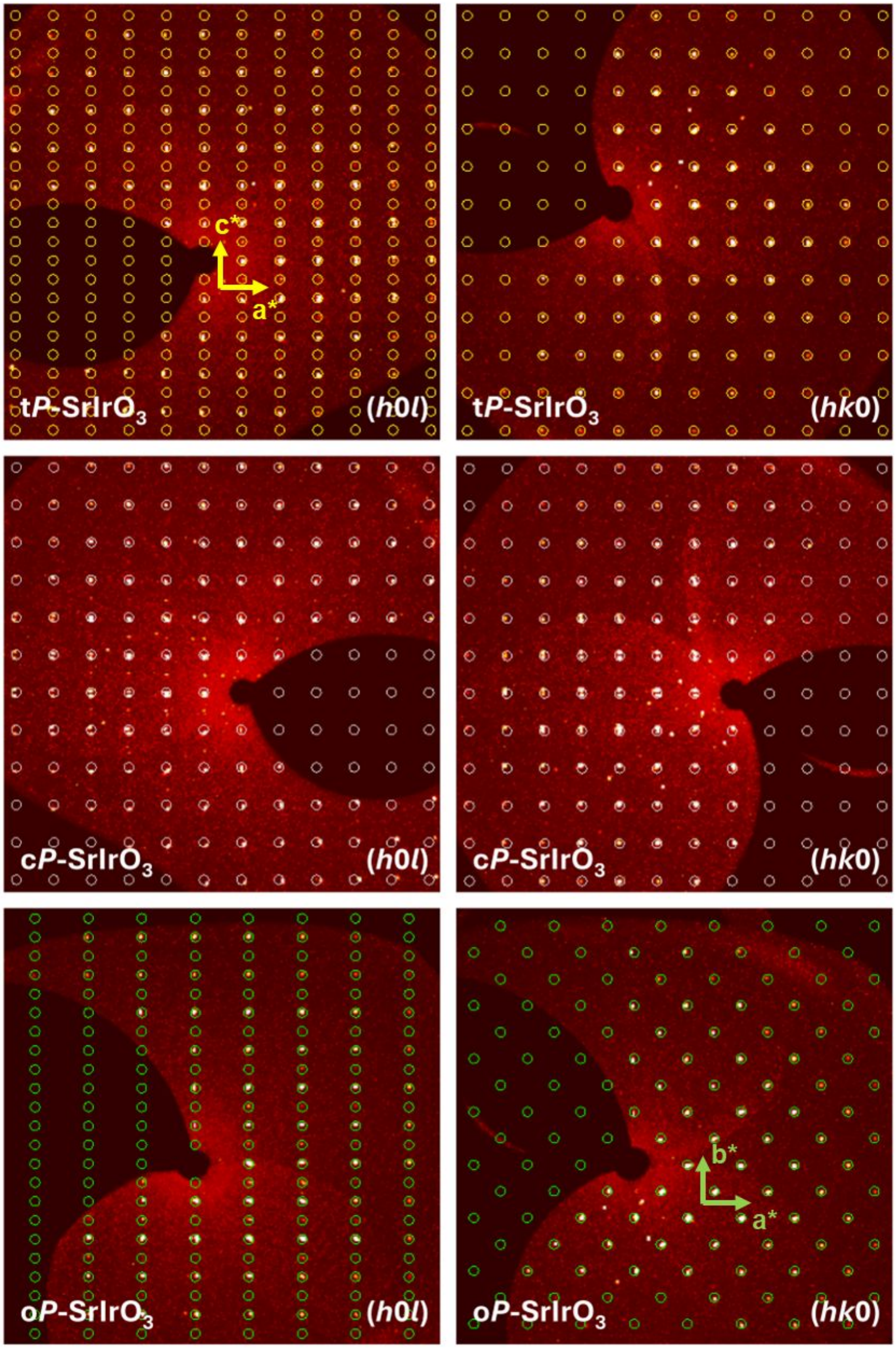

Figure S2 SEM and EDS analysis of a single crystal sample. The formula obtained was calibrated to Ir and listed in the figure.

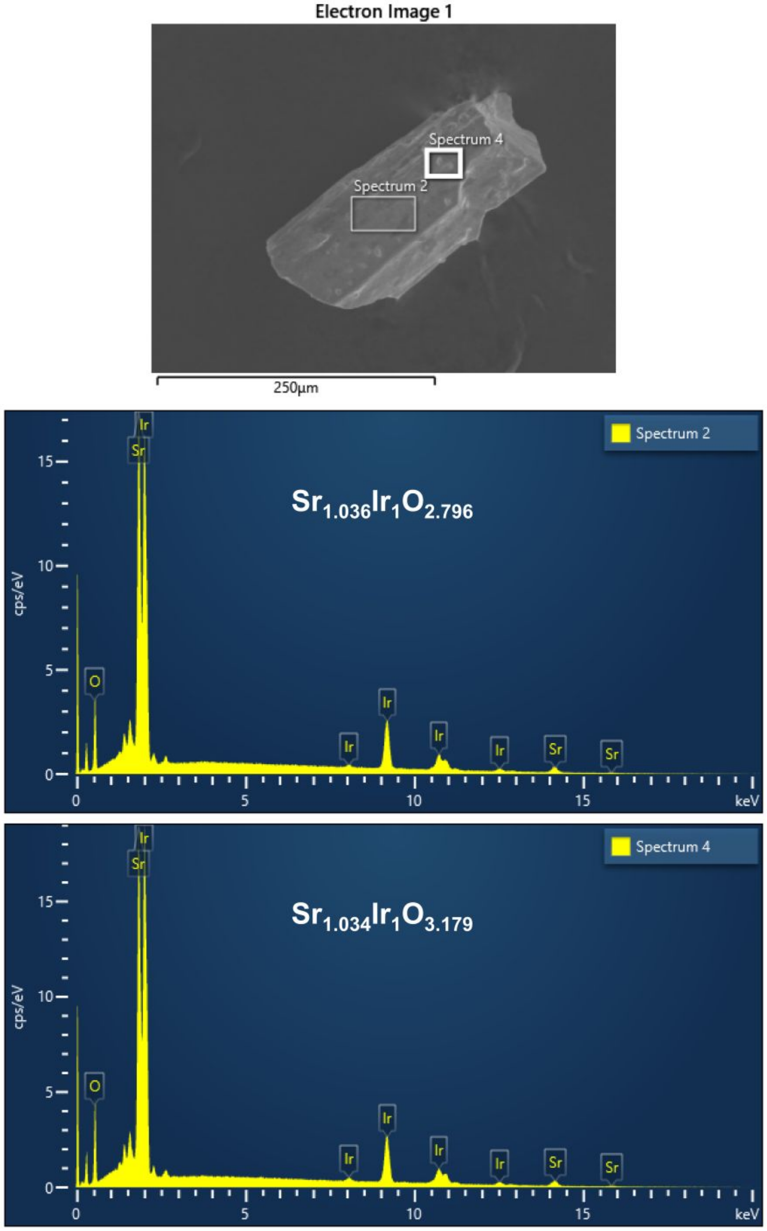

Figure S3 EDS mapping analysis of a single crystal sample. This confirms the homogenous chemical element distribution of the sample.

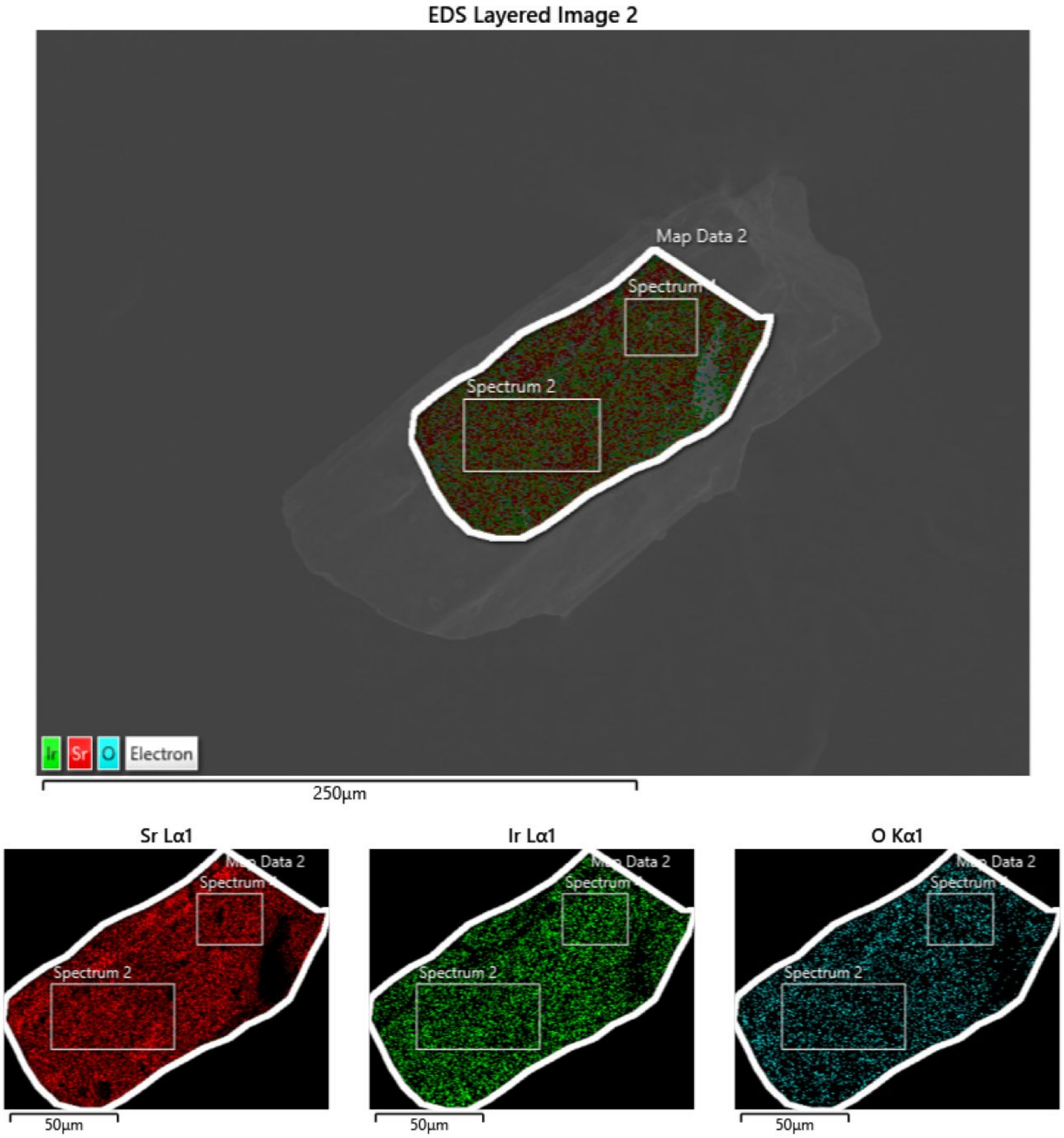

Figure S4 Zoom-in powder XRD Rietveld refinements of t*P*-$SrIrO_3$ and o*P*-$SrIrO_3$.

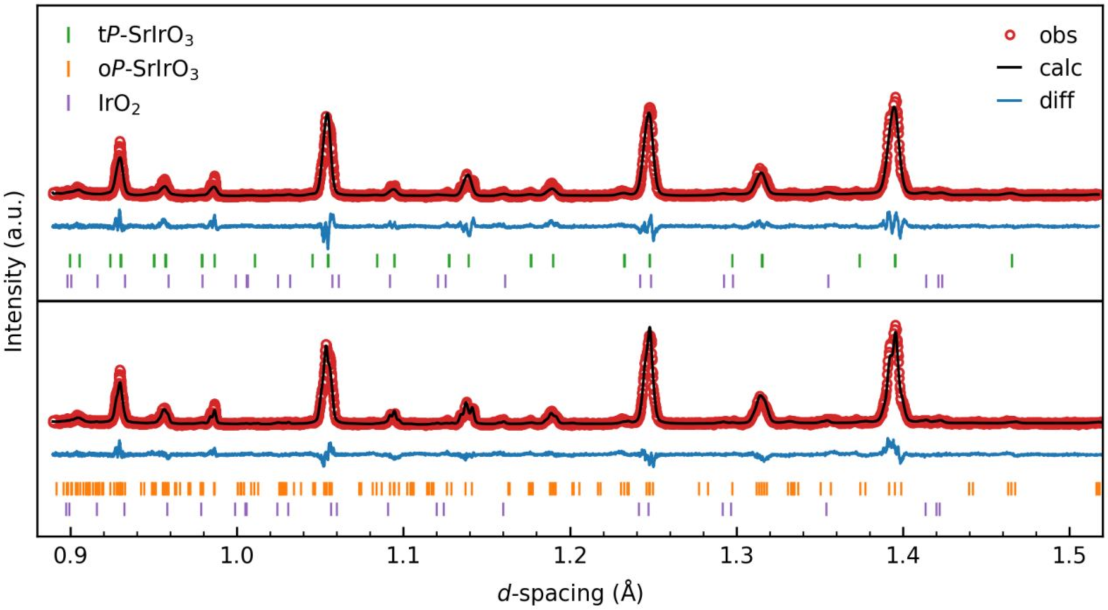

Figure S5 Temperature-dependent magnetic susceptibility in ZFC and FC modes at 1000 Oe. No significant split between ZFC and FC curves was observed.

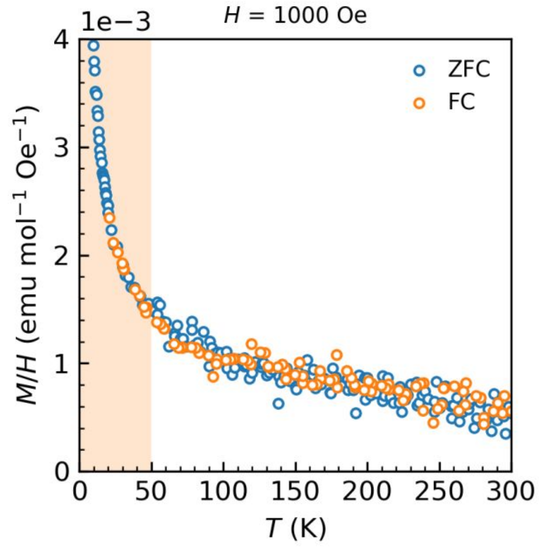

Figure S6 Temperature-dependent electrical resistivity of t*P*-$SrIrO_3$ in different cooling and warming modes.

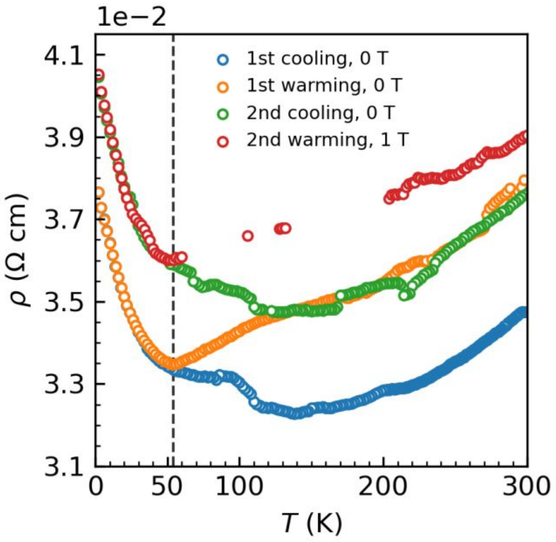

Table S1 Structure parameters from powder XRD Rietveld refinement of m*C*-$SrIrO_3$.

**Compound**	**m*C*-$SrIrO_3$**
Symmetry	*C*2/*c*
Unit cell dimensions	a = 5.60047(27) Å
	b = 9.6254(4) Å
	c = 14.1673(8) Å
	β = 93.1816(28)°
Volume	762.53(8) $Å^3$
wR	13.796%
Goodness-of-fit	2.11
Reduced χ^2	4.46

Table S2 Atomic coordinates and equivalent isotropic atomic displacement parameters ($Å^2$). (U_{eq} is defined as one third of the trace of the orthogonalized U_{ij} tensor.) U_{eq} values of all the oxygen atoms were constrained to be equal.

**m*C*-$SrIrO_3$**	**Wyck.**	***x***	***y***	***z***	**Occ.**	U_{eq}
**Ir_1**	4*a*	0	0	0	1	0.0301(13)
**Ir_2**	8*f*	0.9803(4)	0.66351(31)	0.84596(13)	1	0.0347(8)
**Sr_1**	4*e*	0	0.9980(9)	1/4	1	0.0436(26)
**Sr_2**	8*f*	0.0107(7)	0.6655(7)	0.09845(27)	1	0.0382(17)
**O_1**	4*e*	0	0.570(4)	1/4	1	0.065(5)
**O_2**	8*f*	0.203(5)	0.2647(25)	0.2606(16)	1	0.065(5)
**O_3**	8*f*	0.828(4)	0.3653(26)	0.0416(15)	1	0.065(5)
**O_4**	8*f*	0.901(5)	0.157(3)	0.3995(14)	1	0.065(5)
**O_5**	8*f*	0.300(5)	0.415(3)	0.0933(16)	1	0.065(5)

Table S3 Structure parameters from powder XRD Rietveld refinement of m*C*-$SrIrO_3$ + SiO_2.

**Compound**	**m*C*-$SrIrO_3$**
Symmetry	*C*2/*c*
Unit cell dimensions	a = 5.60065(26) Å
	b = 9.6260(4) Å
	c = 14.1681(8) Å
	β = 93.1818(27)°
Volume	762.65(7) Å^3
wR	12.993%
Goodness-of-fit	1.99
Reduced χ^2	3.96
Weight fraction	m*C*-$SrIrO_3$: 0.928
	SiO_2: 0.072

Table S4 Atomic coordinates and equivalent isotropic atomic displacement parameters (Å^2). (U_{eq} is defined as one third of the trace of the orthogonalized U_{ij} tensor.) U_{eq} values of all the oxygen atoms were constrained to be equal.

**m*C*-$SrIrO_3$**	**Wyck.**	x	y	z	**Occ.**	U_{eq}
**Ir_1**	4*a*	0	0	0	1	0.0312(13)
**Ir_2**	8*f*	0.9804(4)	0.66219(30)	0.84618(14)	1	0.0360(8)
**Sr_1**	4*e*	0	0.0008(9)	1/4	1	0.0374(30)
**Sr_2**	8*f*	0.0110(7)	0.6680(6)	0.09820(26)	1	0.0317(19)
**O_1**	4*e*	0	0.560(4)	1/4	1	0.071(5)
**O_2**	8*f*	0.214(4)	0.2655(24)	0.2594(15)	1	0.071(5)
**O_3**	8*f*	0.827(4)	0.3692(25)	0.0411(14)	1	0.071(5)
**O_4**	8*f*	0.897(5)	0.145(3)	0.3950(13)	1	0.071(5)
**O_5**	8*f*	0.283(5)	0.415(3)	0.0917(16)	1	0.071(5)

Table S5 Crystallographic data and single crystal XRD refinement of t*P*-$SrIrO_3$.

**Chemical Formula**	**$SrIrO_3$**
Formula weight	327.82 g/mol
Space Group	$P4/mmm$
Unit cell dimensions	a = 3.9362(9) Å
	c = 7.880(3) Å
Volume	122.09(7) $Å^3$
Density (calculated)	8.917 g/cm^3
Absorption coefficient	76.005 mm^{-1}
$F(000)$	278
2θ range	5.16 to 79.32°
Total Reflections	1131
Independent reflections	243 [R_{int} = 0.0438]
Refinement method	Full-matrix least-squares on F^2
Data / restraints / parameters	243 / 0 / 18
Final R indices	R_1 (I>2$\sigma(I)$) = 0.0596; wR_2 (I>2$\sigma(I)$) = 0.1247
	R_1 (all) = 0.0648; wR_2 (all) = 0.1289
Largest diff. peak and hole	+14.926 e/$Å^{-3}$ and -12.448 e/$Å^{-3}$
R.M.S. deviation from mean	1.467 e/$Å^{-3}$
Goodness-of-fit on F^2	1.122

Table S6 Atomic coordinates and equivalent isotropic atomic displacement parameters ($Å^2$). (U_{eq} is defined as one third of the trace of the orthogonalized U_{ij} tensor.)

**t*P*-$SrIrO_3$**	**Wyck.**	***x***	***y***	***z***	**Occ.**	**U_{eq}**
**Ir**	2*g*	0	0	0.25009(13)	1	0.0021(4)
**Sr_1**	1*d*	1/2	1/2	1/2	1	0.0002(6)
**Sr_2**	1*c*	1/2	1/2	0	1	0.053(4)
**O_1**	4*i*	0	1/2	0.248(10)	1	0.09(2)
**O_2**	1*b*	0	0	1/2	1	0.11(7)
**O_3**	1*a*	0	0	0	1	0.07(4)

Table S7 Crystallographic data and single crystal XRD refinement of c*P*-$SrIrO_3$.

**Chemical Formula**	**$SrIrO_3$**
Formula weight	327.82 g/mol
Space Group	*Pm*-3*m*
Unit cell dimensions	a = 3.9403(6) Å
Volume	61.18(3) Å^3
Density (calculated)	8.898 g/cm^3
Absorption coefficient	75.841 mm^{-1}
$F(000)$	139
θ range	5.174 to 39.622°
Total Reflections	510
Independent reflections	60 [R_{int} = 0.0389]
Refinement method	Full-matrix least-squares on F^2
Data / restraints / parameters	60 / 0 / 6
Final R indices	R_1 (I>2$\sigma(I)$) = 0.0215; wR_2 (I>2$\sigma(I)$) = 0.0537
	R_1 (all) = 0.0215; wR_2 (all) = 0.0537
Largest diff. peak and hole	+2.835 e/Å^{-3} and -5.662 e/Å^{-3}
R.M.S. deviation from mean	0.594 e/Å^{-3}
Goodness-of-fit on F^2	1.329

Table S8 Atomic coordinates and equivalent isotropic atomic displacement parameters (Å^2). (U_{eq} is defined as one third of the trace of the orthogonalized U_{ij} tensor.)

**c*P*-$SrIrO_3$**	**Wyck.**	***x***	***y***	***z***	**Occ.**	**U_{eq}**
**Ir**	1*b*	1/2	1/2	1/2	1	0.0021(3)
**Sr**	1*a*	0	0	0	1	0.0131(5)
**O**	3*c*	1/2	1/2	0	1	0.091(12)

Table S9 Structure parameters from powder XRD Rietveld refinement of t*P*-$SrIrO_3$ + IrO_2.

**Compound**	**t*P*-$SrIrO_3$**
Symmetry	*P*4/*mmm*
Unit cell dimensions	*a* = 3.94447(22) Å
	c = 7.8938(8) Å
Volume	122.818(6) Å^3
wR	16.957%
Goodness-of-fit	2.37
Reduced χ^2	5.62
Weight fraction	t*P*-$SrIrO_3$: 0.971
	IrO_2: 0.029

Table S10 Atomic coordinates and equivalent isotropic atomic displacement parameters (Å^2). (U_{eq} is defined as one third of the trace of the orthogonalized U_{ij} tensor.)

**t*P*-$SrIrO_3$**	**Wyck.**	***x***	***y***	***z***	**Occ.**	$\boldsymbol{U_{eq}}$
**Ir**	2*g*	0	0	0.24197(18)	1	0.00034(24)
**Sr_1**	1*d*	1/2	1/2	1/2	1	0.0110
**Sr_2**	1*c*	1/2	1/2	0	1	0.0278(11)
**O_1**	4*i*	0	1/2	0.2824(22)	1	0.0235
**O_2**	1*b*	0	0	1/2	1	0.0235
**O_3**	1*a*	0	0	0	1	0.0235

Table S11 Structure parameters from powder XRD Rietveld refinement of c*P*-$SrIrO_3$ + IrO_2.

**Compound**	**c*P*-$SrIrO_3$**
Symmetry	*Pm*-3*m*
Unit cell dimensions	*a* = 3.94478(7) Å
Volume	61.386(3) Å^3
wR	17.524%
Goodness-of-fit	2.45
Reduced χ^2	6.00
Weight fraction	c*P*-$SrIrO_3$: 0.974
	IrO_2: 0.026

Table S12 Atomic coordinates and equivalent isotropic atomic displacement parameters (Å^2). (U_{eq} is defined as one third of the trace of the orthogonalized U_{ij} tensor.)

**c*P*-$SrIrO_3$**	**Wyck.**	***x***	***y***	***z***	**Occ.**	U_{eq}
**Ir**	1*a*	0	0	0	1	0.00153(24)
**Sr**	1*b*	1/2	1/2	1/2	1	0.0162(5)
**O**	3*d*	1/2	0	0	1	0.0191(21)

Table S13 Structure parameters from powder XRD Rietveld refinement of o*P*-$SrIrO_3$ + IrO_2.

**Compound**	**o*P*-$SrIrO_3$**
Symmetry	*Pnma*
Unit cell dimensions	*a* = 5.59521(15) Å
	b = 7.89005(25) Å
	c = 5.56572(16) Å
Volume	245.707(9) $Å^3$
wR	15.296%
Goodness-of-fit	2.14
Reduced χ^2	4.58
Weight fraction	o*P*-$SrIrO_3$: 0.974
	IrO_2: 0.026

Table S14 Atomic coordinates and equivalent isotropic atomic displacement parameters ($Å^2$). (U_{eq} is defined as one third of the trace of the orthogonalized U_{ij} tensor.)

**o*P*-$SrIrO_3$**	**Wyck.**	***x***	***y***	***z***	**Occ.**	**U_{eq}**
**Ir**	4*b*	1/2	0	0	1	0.00144(22)
**Sr**	4*c*	0.5251(5)	1/4	0.4838(7)	1	0.0081(5)
**O_1**	4*c*	0.510(4)	1/4	0.995(11)	1	0.0293
**O_2**	8*d*	0.273(4)	0.016(4)	0.729(8)	1	0.0293
